# Cost to implement an outpatient stewardship intervention for acute otitis media (AOM)

**DOI:** 10.1017/ash.2026.10428

**Published:** 2026-07-01

**Authors:** Leisha M. Andersen, Sophie E. Katz, Amy Keith, Theresa L. Morin, Timothy C. Jenkins, Alexander S. Plattner, Evan Facer, Sherry Dodd, Sharon Graham, Holly M. Frost, for the RELAX Study Group

**Affiliations:** 1 American Academy of Pediatrics, USA; 2 Vanderbilt University Medical Center, USA; 3 Denver Health and Hospital Authority, USA; 4 Intermountain Health, USA; 5 Washington University in St Louis, USA

## Abstract

**Objective:**

Most children 2 years and older with uncomplicated acute otitis media (AOM) are prescribed 10-day antibiotic durations, despite national guidelines recommending antibiotics for 5–7 days. Costs are often cited as a barrier to stewardship efforts. As part of a larger clinical trial including 2 systems and 46 clinics, we developed a low-intensity and a high-intensity intervention aimed at reducing antibiotic duration for AOM and evaluated implementation and sustainability for the interventions.

**Methods:**

Costs associated with each implementation activity were recorded over time, including material/supply costs (eg, printing) and personnel time costs. Sustainability costs were estimated based on ongoing implementation expenses. For each system, we assessed total intervention, activity-specific, and sustainability costs. Aggregate results were reported as the median across systems.

**Results:**

The total median implementation costs were $3,606 (range $2,540–$4,672) for the low-intensity intervention and $9,203 (range $7,557–$10,849) for the high-intensity intervention. For the low-intensity intervention, the primary cost driver was electronic health record modifications totaling $2,292 (range $1,615–$2,968). For the high-intensity intervention, the primary cost driver was audit and feedback system activation totaling $5,597 (range $2,885–$8,309). Personnel time accounted for over 90% of costs in both study arms. Sustainability costs were $133/year (range $77–$190) for the low-intensity intervention and $764/year (range $628–$901) for the high-intensity intervention.

**Conclusions:**

Overall costs were low. The high-intensity intervention resulted in higher costs compared to the low-intensity intervention.

## Introduction

Acute otitis media (AOM) affects 60% of children by 3 years of age and accounts for 24% of all pediatric antibiotic prescriptions.^1,2^ The 2013 American Academy of Pediatrics Clinical Practice Guidelines recommend short antibiotic (5–7 d) durations for most children 2 years of age and older meeting criteria for antimicrobial management.^3^ Compared to 10-day antibiotic durations, shorter durations are similarly effective, reduce the incidence of adverse drug events, and are viewed favorably by both clinicians and parents.^4–6^ Optimizing prescribing practices is central to reducing antibiotic overuse for AOM and other common pediatric conditions associated with excessive prescribing (eg, pharyngitis, viral upper respiratory tract infections).^7^ Although antibiotic stewardship programs have been shown to be effective for improving prescribing practices and reducing antibiotic overuse, the costs associated with intervention activation are poorly understood, and the financial demands of program activation are often cited as a barrier to implementation.^8,9^

Prior interventions (eg, clinician education; clinical decision support) have successfully increased prescribing of recommended short durations for uncomplicated AOM in a variety of pediatric healthcare settings (emergency departments, urgent cares, primary care clinics).^10–12^ Interventions based on the Centers for Disease Control and Prevention (CDC) Core Elements of Outpatient Antibiotic Stewardship (commitment, action for policy and practice, clinician feedback, and education/expertise) have also shown success in increasing prescribing of guideline-concordant antibiotic durations with improved or similar patient outcomes.^12–15^ Despite these successes, generalized adoption of short-duration prescribing is lacking. In fact, nationwide claims data show that over 90% of AOM prescriptions continue to be written for 10 days.^16^ To encourage guideline-concordant prescribing in children, health systems must weigh the costs of intervention implementation and maintenance against the risks of incurring antibiotic exposure-associated consequences.

To understand the optimal method for increasing guideline-concordant, short-duration prescribing, we conducted a multicenter cluster-randomized clinical trial based on the CDC recommendations and compared the effectiveness and implementation outcomes of a low-intensity and high-intensity intervention. In this sub-analysis, we aimed to describe the direct (material/supply) and indirect (personnel time) costs associated with implementation and sustainability of the low- and high-intensity interventions. The goal of this project was to understand the resources likely needed for other health systems to implement and sustain the interventions.

## Methods

### RELAX program implementation

The Reducing Length of Antibiotics for Children with Ear Infections (RELAX) Trial used a 2-year multicenter cluster-randomized design to compare the effectiveness of a low- and high-intensity intervention to increase prescribing of short (5-d) antibiotic durations for uncomplicated AOM in children ≥2 years of age. The interventions were based on the CDC Core Elements of Outpatient Antibiotic Stewardship.^15^ In total, the RELAX system-level intervention was implemented at 46 outpatient pediatric clinics at Vanderbilt University Medical Center (VUMC; 35 clinics; primary care (4), urgent care (16), retail (15)) and Washington University (WashU; 11 clinics; all primary care). The sites represented the broader communities and included a diverse payer mix. Characteristics of the systems have been previously published by Keith et al (2023).^17^

The project consisted of a 12-month preimplementation period (April 2023–April 2024) followed by a 24-month implementation period (April 2024–April 2026). During the preimplementation period, clinics were randomized to receive 1 of 2 interventions. The low-intensity intervention consisted of clinician champions, patient education, clinician education, and electronic health record (EHR) system modifications including prescription field changes and access to clinical care guidelines. The high-intensity intervention consisted of all low-intensity intervention components in addition to individualized audit and feedback prescribing reports using the Outpatient Automated Stewardship Information System statistical code with a read receipt tracking mechanism. Systems were encouraged to adapt intervention components to accommodate site-specific context and needs throughout the preimplementation and implementation periods.^18^ Adaptations were recorded and analyzed. Table 1 describes the intervention components, implementation activities, and system-specific action steps and adaptations.

**Table 1.** Intervention components, implementation strategies, and system-specific action steps and strategy adaptations Table 1 long description.

Intervention component	Implementation strategy	System-specific action steps and strategy adaptations	
		VUMC actions	WashU actions
**Clinician champion(s) identification** ^a^	Identify 1 or more clinician champions at each system	Identified 1 clinician champion	Identified 3 clinician champions
**Patient-facing educational resource adaptation and dissemination** ^b^	IAdapt and disseminate patient-facing open-access educational resources to participating clinicians	IAdapted open-access resource (with input from system-specific patient education team) Translated into Spanish and Arabic Printed (handout format) Distributed to walk-in clinics and 1 primary care site Created smart-phrase to promote resource inclusion in patient after-visit summaries	Printed open-access resource (poster format) Distributed poster to all participating clinical sites within the system
**Clinician-facing educational resource adaptation, dissemination, and credit tracking** ^c,d^	Adapt and disseminate clinician-facing open-access educational resource Market learning sessions Track and attest to learning session participation	Distributed open-access resource to all participating clinicians Printed smart-phrase educational resource (poster format) Distributed smart-phrase educational resource to all participating clinical sites Distributed learning session invitation and credit eligibility information to all participating clinicians Tracked and attested to participant learning session participation	Distributed open-access resource to all participating clinicians Distributed learning session invitation and credit eligibility information to all participating clinicians Tracked and attested to participant learning session participation
**Electronic health record (EHR) modification and activation** ^e^	System-specific refinement of the EHR based on core team recommendations (eg, adding hyperlinks to local clinical care guidelines, adding “help” text to guide real-time prescribing, and adding patient education materials to the EHR) and activation of refined EHR	Gained leadership approval for proposed EHR changes (from antimicrobial stewardship committee) Notified clinicians about EHR changes	Gained leadership approval for proposed EHR changes (from pharmacy stakeholders, clinical decision support team, and Chief Medical Informatics Officer)
**Individualized audit and feedback system activation** ^f^ *For high-intensity participants only	Creation of individualized clinician feedback reports based on the Outpatient Automated Stewardship Information System^15^ statistical code with a read receipt tracking mechanism Quarterly dissemination of reports to high-intensity participants Quarterly tracking of read receipts	Created feedback system Disseminated quarterly reports Tracked read receipts for quarterly reports	Modified existing feedback system Disseminated quarterly report dissemination Tracked read receipts for quarterly reports

*Note*. VUMC, Vanderbilt University Medical Center; WashU, Washington University.

a Clinicians champions were designated and charged with liaising between the core team and the system-specific teams to transmit communications, respond to questions, etc.

b Patient-facing electronic/printable PDF educational resources (brochure and poster) described the rationale for short-duration antibiotic prescribing for eligible patients with acute otitis media (AOM).

c Clinician-facing electronic/printable PDF educational resource (brochure) described the evidence for managing eligible AOM infections with national guideline-concordant short-duration prescribing.

d Live/virtual learning sessions (2 × 60 min) reviewed the evidence for 5–7 day antibiotic durations for eligible AOM patients and introduced communications strategies (Continuing Medical Education and Maintenance of Certification, Part 2 credit available for clinician participation).

e Adaptations to the EHR included suggestions to reset prescription field antibiotic default durations to 5 days, add hyperlinks to local clinical care guidelines, add “help” text to guide real-time prescribing, and add linkages to patient-facing educational resources.

f Quarterly individualized feedback reports showed the proportion of individual-level antibiotic prescriptions written for the recommended 5-day duration for children ≥2 years of age with AOM (including peer comparisons).

Notably, clinicians received educational credit for participation in each of the 2 learning sessions (Continuing Medical Education, 1.0 credit CME, and American Board of Pediatrics [ABP] Maintenance of Certification [MOC] Part 2, 1.0-point MOC Part 2). In addition, pediatric clinicians received ABP MOC Part 4 credit (25 points MOC, Part 4 for each of 2 action periods) for active project participation for at least 6 months, attendance at all required system-specific meetings, attendance at all learning sessions, completion of the postparticipation survey, and review of at least 1 cycle of project data (for high-intensity participants only). Family Medicine clinicians received American Board of Family Medicine Performance Improvement credit (20 points ABFM PI) for project participation (including planning and executing tests of change) and data review. Details of the intervention protocol and constructs were previously described by Keith et al.^17^

### Cost processing and analysis

We used time-driven, activity-based costing (also called micro-costing) to assess intervention-associated expenses.^19,20^ For each implementation activity, system leads recorded all material/supply and personnel time costs associated with the action step. Preimplementation costs associated with the development of tools and educational opportunities (eg, educational resources, CME and MOC accrediting) were excluded from the costing analysis because the tools were designed to be open access and free for use and adaptation by future health systems. Further, both CME and MOC implementation costs are typically absorbed at the system level and are anticipated to be negligible. Time and costs specifically associated with the conduct of research, but not the intervention components or implementation activities, were also excluded because the objective was to understand the estimated costs of implementing the antibiotic stewardship intervention components at other health systems.

To guide systems in the process of micro-costing, we created a standardized cost template worksheet adapted from the Cost Of Implementing New Strategies (COINS) framework (Supplemental Table 4).^20^ The standardized worksheet outlined the intervention components and implementation activities, assigned to 6 broad categories: (1) clinical champion identification; (2) patient-facing educational resource adaptation and dissemination; (3) clinician-facing educational resource adaptation, dissemination, and credit tracking; (4) EHR prescription field modification and activation; (5) individualized audit and feedback system creation and activation; and (6) general costs. For each outlined implementation activity, the worksheet prompted entry of expenses for all system-specific action steps and adaptations, including the date of action, specific action taken, person performing the action, associated material/supply costs, and associated personnel time (hours) allocated to completion of the task.

During the preimplementation period, the standardized cost template worksheet was presented to system leads for feedback. Once finalized, system leads received online access to a system-specific cost worksheet and training for completing the form. Training included instructions to document material/supply and personnel time allocations in real-time throughout the implementation period to reduce recall bias. Each system’s cost worksheet was reviewed by the core study team quarterly throughout the implementation period and verified with system leads via email or regularly scheduled monthly meetings to confirm data accuracy. Sixteen months into the 24-month implementation period, implementation costs were assessed, and system leads were asked to predict sustainability costs based on ongoing expenses since implementation.

The completed standardized cost worksheet data were used to tabulate activity-specific and aggregate cost estimates for each system. Interventions were completed at the system level, and all costs reflect system-level rather than clinic-level expenses. Material/supply costs were summed by action step and activity. Personnel time costs were assigned an expense by multiplying hours allocated to the task by the median hourly wage of the personnel performing the activity. US Bureau of Labor Statistics (BLS) national median values (aligned with the individual’s position title and degree) were used for hourly wage estimates to normalize for geographic variability.^21^ Wage estimates included fringe rates (as assigned by the BLS at 38.4%) and were not discounted or adjusted for time given the short duration of the study. BLS median hourly wages used for personnel time cost estimates are summarized (Supplemental Table 2 and 3).

**Table 2.** Costs by intervention component, implementation strategy, and system Table 2 long description.

Intervention component and implementation strategy	VUMC			WashU			MEDIAN		
	Material/supply costs $^a^	Personnel time cost in $ (personnel hours allocated to task * BLS median hourly wage for assigned personnel^b^)	Total cost, $	Material/supply costs $^a^	Personnel time cost in $ (personnel hours allocated to task * BLS median hourly wage for assigned personnel^b^)	Total cost, $	Median material/supply costs $^a,c^	Median personnel time cost in $^c^	Median total cost, $^c^
**Clinician champion identification**	**0**	**0**	**0**	**0**	**0**	**0**	**0**	**0**	**0**
**Patient-facing educational resource adaptation and dissemination**	**150**	**979**	**1,129**	**451**	**332**	**783**	**301**	**656**	**956**
System-specific adaptations	0	24	24	0	95	95	0	60	60
Paper and printing	150	0	150	399	47	446	275	24	299
Resource distribution within system	0	955	955	52	190	242	26	573	599
**Clinician-facing educational resource adaptation, dissemination, and credit tracking**	**39**	**536**	**575**	**0**	**142**	**142**	**20**	**339**	**359**
System-specific adaptations	0	96	96	0	0	0	0	48	48
Paper and printing	39	0	39	0	0	0	20	0	20
Resource distribution to clinics within system	0	96	96	0	47	47	0	72	72
Learning session marketing	0	191	191	0	0	0	0	96	96
Learning session credit notification and attestation (CME/MOC2/MOC4/ABFM PI)	0	153	153	0	95	95	0	124	124
**Electronic health record (EHR) prescription field modification and activation**	**0**	**2,968**	**2,968**	**0**	**1,615**	**1,615**	**0**	**2,292**	**2,292**
Ensuring system-specific approval for EHR changes	0	812	812	0	955	955	0	884	884
Oversight of on-site IT build with modifications (eg, prescription field changes)	0	1,487	1,487	0	229	229	0	858	858
Activation of new EHR build and notification of clinicians	0	669	669	0	431	431	0	550	550
**Individualized audit and feedback system creation and activation** *For use by high-intensity arm only	**0**	**2,885**	**2,885**	**0**	**8,309**	**8,309**	**0**	**5,597**	**5,597**
Code establishment and beta testing	0	1,393	1,393	0	7,165	7,165	0	4,279	4,279
Creation of report distribution system	0	50	50	0	286	286	0	168	168
Education of clinicians about reports	0	191	191	0	572	572	0	382	382
Report activation with quarterly tracking of read receipts	0	1,251	1,251	0	286	286	0	769	769
**Total costs low-intensity**	**189**	**4,483**	**4,672**	**451**	**2,089**	**2,540**	**320**	**3,286**	**3,606**
**Total costs high-intensity**	**189**	**7,368**	**7,557**	**451**	**10,398**	**10,849**	**320**	**8,883**	**9,203**

*Note.* VUMC, Vanderbilt University Medical Center; WashU, Washington University; CME, Continuing Medical Education; MOC, Maintenance of Certification; ABFM PI, American Board of Family Medicine Performance Improvement.

a All costs presented in 2025 USD.

b National fringe rate 38.4% from the Bureau of Labor Statistics (BLS).^21^

c Total median values may differ from actual total values due to rounding error.

**Table 3.** Personnel time costs (hours) by intervention component, implementation strategy, and system Table 3 long description.

	VUMC	WashU	MEDIAN
Intervention component and implementation strategy	Personnel time costs (hours)	Personnel time costs (hours)	Median personnel time costs (hours)
**Clinician champion identification**	**0**	**0**	**0**
**Patient-facing educational resource adaptation and dissemination**	**10.25**	**7**	**8.63**
System-specific adaptations	0.25	2	1.1
Paper and printing	0	1	0.5
Resource distribution within system	10	4	7
**Clinician-facing educational resource adaptation, dissemination, and credit tracking**	**8**	**3**	**5.5**
System-specific adaptations	1	0	0.5
Paper and printing	0	0	0
Resource distribution to clinics within system	1	1	1
Learning session marketing	2	0	1
Learning session credit notification and attestation (CME/MOC2/MOC4/ABFM PI)	4	2	3
**Electronic health record (EHR)** **prescription field modification and activation**	**37**	**17.75**	**27.38**
Ensuring system-specific approval for EHR changes	8	10	9
Oversight of on-site IT build with modifications (eg, prescription field changes)	22	3	12.5
Activation of new EHR build and notification of clinicians	7	4.75	5.8
**Individualized audit and feedback system creation and activation** *For use by high-intensity arm only	**55**	**91**	**73**
Code establishment and beta testing	28	75	51.5
Creation of report distribution system	1	4	2.5
Education of clinicians about reports	2	8	5
Report activation with quarterly tracking of read receipts	24	4	14
**Total personnel hours low-intensity arm**	**55.25**	**27.75**	**41.5**
**Total personnel hours high-intensity arm**	**110.25**	**118.75**	**114.5**

*Note.* VUMC, Vanderbilt University Medical Center; WashU, Washington University; CME, Continuing Medical Education; MOC, Maintenance of Certification; ABFM PI, American Board of Family Medicine Performance Improvement.

From each system’s completed cost worksheet, a calculation of the material/supply and personnel time costs associated with completion of each action step was made. Action step costs were then summed by implementation activity and aggregated by intervention component. Following the initial calculations, the results were returned to the system leads for review and input prior to finalization. Once finalized, costs were compared across intervention components and across implementation systems, and median values were derived for each element. Costs associated with the low- and high-intensity intervention components were assessed separately. The costs, including differential costs between low- and high-intensity intervention arms, could serve as an estimate of the material/supply and personnel time cost to replicate the low- and high-intensity RELAX programs at future systems.

The RELAX study (NCT05608993) was reviewed by the Colorado Multiple Institutional Review Board at the University of Colorado in Denver. The study was deemed minimal risk and was granted an exemption from obtaining informed consent for the use of the secondary data included in the analysis.

## Results

System-level costs for each intervention component and implementation activity are shown in Table 2.

### Total costs

For the low-intensity intervention, the median total implementation cost per system was $3,606 (range $2,540–$4,672). Median material/supply cost was $320 (range $189–$451). Median personnel time cost was $3286 (range $2,089–$4,483). For the high-intensity intervention, the median total implementation cost per system was $9,203 (range $7,557–$10,849). Median material/supply cost was $320 (range $189–$451). Median personnel time cost was $8,883 (range $7,368–$10,398).

### Intervention costs by activity

Activity-associated costs for each intervention component are shown in Table 2. For the low-intensity intervention, the activity with the highest overall expense was EHR modification and activation with a median cost of $2,292 (range $1,615–$2968), and all costs were attributed to personnel time. Other activity-associated costs in the low-intensity arm included: clinician champion identification (median cost $0), patient-facing educational resource adaptation and dissemination (median cost $956, range $783–$1,129), and clinician-facing educational resource adaptation, dissemination, and credit tracking (median cost $359, range $142–$575). For the high-intensity intervention, the activity with the highest overall expense was individualized audit and feedback system creation and activation with a median cost of $5,597 (range $2,885–$8,309), with all costs attributed to personnel time.

### Personnel time expenditures

Personnel time costs for each intervention component are shown in Table 3 and Supplemental Tables 1 and 3. For the low-intensity intervention, the median personnel time per system (in hours) for implementation was 41.5 (range 27.75–55.25). Of the total expended personnel hours, 55% were allocated to clinicians, 26% to pharmacists, 17% to project or operation managers, and 2% to data or informational scientists. For the high-intensity intervention, the median personnel time for implementation was 114.5 (range 110.25–118.75). Of the total expended personnel hours, 58% were allocated to clinicians, 20% to data or informational scientists, 13% to project or operation managers, and 9% to pharmacists.

### Sustainability costs

Sustainability costs for each intervention component are shown in Table 4. For the low-intensity intervention, the median annual sustainability cost was $133 (range $77–$190), with all costs attributed to personnel time. For the high-intensity intervention, the median annual sustainability cost was $764 (range $628–$901), with all costs attributed to personnel time.

**Table 4.** Cost by intervention sustainability component and system Table 4 long description.

Sustainability component	VUMC			WashU			Total median personnel hours by intervention component (hours)^c^	Total median cost by intervention component ($)^c^
	Material/supply costs $^a^	Personnel time costs^b^ (hours)	Personnel time cost in $ (personnel hours allocated to task * BLS median hourly wage for assigned personnel)	Material/supply costs $^a^	Personnel time costs^b^ (hours)	Personnel time cost in $ (personnel hours allocated to task * BLS median hourly wage for assigned personnel)		
Clinician champions	0	0	0	0	0	0	0	0
Patient educational resources	0	0	0	0	0	0	0	0
Clinician educational resources	0	0	0	0	4	190	2	95
Electronic health record (EHR) prescription field modification	0	2	77	0	0	0	1	38
Individualized audit and feedback report creation and quarterly distribution	0	12	551	0	14.5	711	13.25	631
Total costs low-intensity arm	0	2	77	0	4	190	3	133
Total costs high-intensity arm	0	14	628	0	18.5	901	16.25	764

*Note.* VUMC, Vanderbilt University Medical Center; WashU, Washington University.

a All costs presented in 2025 USD.

b National fringe rate 38.4% from the Bureau of Labor Statistics (BLS).^21^

c Total median values may differ from actual total values due to rounding error.

## Discussion

In this study, we implemented 2 low-cost, system-level interventions aimed at improving the adoption of short-duration prescribing for uncomplicated AOM. Most implementation expenses were attributable to EHR modifications (low-intensity arm) and audit and feedback system activation (high-intensity arm). Sustainability costs were minimal for the studied systems as enduring materials are already created and freely available for continued use. Therefore, the current economic evaluation provides practical data to assess feasibility and resource allocation at future antimicrobial stewardship-focused adoption systems, regardless of health system size.

As expected, the high-intensity intervention was more expensive to implement and sustain compared to the low-intensity intervention. The ∼2.5-fold higher implementation cost (and >5-fold sustainability cost) of the high-intensity intervention underscores the resource demand to develop, activate, and maintain audit and feedback systems. As outlined in the CDC Core Elements of Outpatient Antibiotic Stewardship and confirmed by prior research in pediatric primary care clinics, audit and feedback systems promote sustained practice change by increasing clinician awareness of prescribing behavior.^15,22^ With the inclusion of all 4 CDC Core Elements of Antibiotic Stewardship, the high-intensity intervention aligns most closely with best practices and evidence-based recommendations for modifying prescribing practices in outpatient pediatric settings.^15^ However, prior evidence also supports the success of EHR-only interventions to promote uptake of guideline-concordant prescribing.^12^ Resource-limited settings should consider the costs and benefits of establishing audit and feedback systems when determining stewardship components for implementation.

Our use of a time-driven, activity-based cost tracking template improved the precision of the projected resource estimates and helped to document, in real-time, all material/supply and personnel time expenses. Cost tracking results identified personnel hours as the primary cost driver for both the low-intensity and high-intensity intervention arms, mirroring findings from hospital-targeted stewardship programs.^23^ Our study specifically identified clinician-led personnel time as the main contributor to system-based personnel time costs, generating 55% of personnel time costs in the low-intensity arm and 58% of personnel time costs in the high-intensity arm. Leveraging team support is a well-established key component when initiating successful stewardship programming and strategically selecting strong, stewardship-focused clinical champions has the potential to maximize efficiency and contain expenses when activating an outpatient stewardship program.^15,24^

This study filled a notable gap in evidence regarding the cost of implementing and sustaining stewardship interventions in community-based primary care, urgent care, and retail settings. Prior economic modeling primarily targets the costs (eg, salary support for core personnel) and benefits (eg, reduced days of therapy, decline in average antibiotic use, cost per avoided resistance, cost per life years gained) of hospital-based stewardship interventions.^25–27^ With 80% of antibiotics prescribed in outpatient settings and up to 50% of those prescriptions deemed unnecessary or inappropriate, greater emphasis on the long-term value of outpatient pediatric stewardship programs is warranted.^28,29^

This study has several limitations. First, although we evaluated costs at 2 large health systems using USt BLS national hourly wage median estimates to normalize for geographic variability, our findings may not reflect costs at geographically distinct healthcare settings. The use of precise wages could have strengthened the accuracy of the wage estimates for this study. Second, despite requesting real-time documentation of personnel hour data throughout the project, over- or underestimates may have occurred, and reporting bias may have impacted the personnel hour attributions and skewed final cost calculations. Third, the time spent by clinician participants on RELAX activities was not tracked for this project. Fourth, both VUMC and WashU had an underlying stewardship infrastructure in place before project implementation, and both used the EPIC® (Verona, WI) EHR platform with on-site support. Health systems with a less-established stewardship infrastructure (eg, with reduced educational support for CME/MOC or with alternative EHR vendors or support systems) may accrue additional implementation or sustainability expenses. Specifically, sustainability costs, although minimal for the investigated systems, could vary for future implementation sites depending on the scope and resourcefulness of each system’s existing antibiotic stewardship infrastructure. Finally, this report does not present data on the success of the intervention, which may shift the perceived cost-effectiveness. This cost analysis was part of a broader clinical trial, and the study’s impact on antibiotic prescribing will be published at the trial’s conclusion.

## Conclusion

In conclusion, understanding cost is essential to assessing the feasibility of antibiotic stewardship program adoption. Although expense is often cited as a barrier to program uptake, these findings suggest that antimicrobial stewardship activities can be implemented and sustained with low-cost interventions in pediatric outpatient settings.

## Supporting information

10.1017/ash.2026.10428.sm001 Andersen et al. supplementary material Andersen et al. supplementary material

## Data Availability

Data is not publicly available.

## Supplementary material

The supplementary material for this article can be found at https://doi.org/10.1017/ash.2026.10428.

## The RELAX study group

Ivanna V. Bell, Aiden Gilbert, MPA, Hannah Jensen, BS, Sonja O’Leary, MD, Deborah J. Rinehart, PhD, and Gale Thomas.

## Table 1. Long description

The table presents a detailed comparison of intervention components, implementation strategies, and system-specific action steps and strategy adaptations. It includes four main columns: Intervention, Implementation strategy, System-specific action steps and adaptations, and Watch actions. The table has multiple rows, each detailing specific actions and adaptations for different interventions. For instance, the clinician champion identification involves identifying one or more champions at each clinic and has specific watch actions. Patient-facing resources include adapting and disseminating patient-facing open-access educational resources, with system-specific actions like input from system-specific patient education teams. Clinician-facing resources involve adapting and disseminating clinician-facing open-access educational resources, with actions like distributing these resources to participating clinics. The electronic health record system modifications include recommendations like adding hyperlinks to local clinical care guidelines and adding help text to guide real-time prescribing. The high-intensity intervention includes all low-intensity components plus individualized audit and feedback prescribing reports. The table also tracks adaptations and watch actions for each intervention component, providing a comprehensive overview of the implementation process.

Navigate back to Table 1..

## Table 2. Long description

The table presents a detailed comparison of costs associated with various intervention components and implementation strategies across VUMC, WashU, and median values. It includes material/supply costs, personnel time costs, and total costs for different activities such as clinician champion identification, patient-facing educational resource adaptation, system-specific adaptations, and more. The table is organized into multiple rows and columns, with each row representing a specific intervention component and each column representing the costs at VUMC, WashU, and the median values. Notable trends include varying costs for different activities and strategies, with some components showing significantly higher costs at certain institutions. The table provides a comprehensive overview of the financial implications of implementing these interventions.

Navigate back to Table 2..

## Table 3. Long description

The table presents personnel time costs in hours for different intervention components and implementation strategies across two systems, VUMC and WashU, along with median values. It includes rows for clinician champion identification, patient-facing educational resource adaptation and dissemination, clinician-facing educational resource adaptation, dissemination, and credit tracking, electronic health record prescription field modification and activation, and individualized audit and feedback system creation and activation. Each row details specific tasks such as system-specific adaptations, paper and printing, resource distribution, learning session marketing, and more. The table also summarizes total personnel hours for low-intensity and high-intensity arms. Notable trends include varying time costs for tasks across the two systems, with some tasks showing significant differences in hours spent.

Navigate back to Table 3..

## Table 4. Long description

The table compares costs by intervention sustainability component and system, detailing material and personnel time costs for various activities across two systems, VUMC and WashU. It includes columns for material/supply costs, personnel time costs in hours, personnel time cost in dollars, and total median personnel hours and costs by intervention component. The rows list different sustainability components such as clinician champions, patient educational resources, clinician educational resources, electronic health record prescription field modification, and individualized audit and feedback report creation and quarterly distribution. The table shows data for both low-intensity and high-intensity arms, with notable costs in the individualized audit and feedback report creation and quarterly distribution row for both systems. The total costs for the high-intensity arm are significantly higher than those for the low-intensity arm.

Navigate back to Table 4..
