# Supplementary material for: Cost to implement an outpatient stewardship intervention for acute otitis media (AOM)
Source: Antimicrob Steward Healthc Epidemiol. 2026 Jul 1;6(1):e171. doi: 10.1017/ash.2026.10428 (PMC13343337; doi:10.1017/ash.2026.10428)
Supplement: Andersen et al. supplementary material [file S2732494X26104288sup001.docx]

**Supplemental Table 1**. Crosswalk between Low- and High-intensity Intervention Arm Site-based Personnel Time Costs and Percent Total Contribution

| Site 1 Site 2  VUMC WashU | | | | | Median | hours |
| --- | --- | --- | --- | --- | --- | --- |
| Personnel Role | Low-intensity hours (% of total) | High-intensity hours (% of total) | Low-intensity hours (% of total) | High-intensity hours (% of total) | Low-intensity hours (% of total) | High-intensity hours (% of total) |
| Clinician | 31.25 | 36.25 | 14 | 97 | 22.63 | 66.63 |
| (MD) | (57%) | (33%) | (51%) | (82%) | (55%) | (58%) |
| Data or | 0 | 43 | 2 | 2 | 1 | 22.5 |
| informational | (0%) | (39%) | (7%) | (2%) | (2%) | (20%) |
| Scientist |  |  |  |  |  |  |
| Pharmacist | 20 | 20 | 1.75 | 1.75 | 10.88 | 10.88 |
|  | (36%) | (18%) | (6%) | (1%) | (26%) | (9%) |
| Project or | 4 | 11 | 10 | 18 | 7 | 14.5 |
| Operation | (7%) | (10%) | (36%) | (15%) | (17%) | (13%) |
| Manager |  |  |  |  |  |  |
| TOTAL | 55.25 | 110.25 | 27.75 | 118.75 | 41.5 | 114.5 |
| HOURS | (100%) | (100%) | (100%) | (100%) | (100%) | (100%) |

**Supplemental Table 2**. National Wage Estimates

| Site | US Bureau of Labor Statistics link by role | Missouri Median Annual Wage ($) | Tennessee Median Annual Wage ($) | US Bureau of Labor Statistics  Median Annual Wage ($) | Median hourly wage ($) |
| --- | --- | --- | --- | --- | --- |
| VUMC | Pediatrician, General link  (2023) | 204,120 | 189,930 | 198,690 | 95.53 |
|  | General Internal Medicine Physicians  link (2023) | 228,030 | 64,590 | 223,310 | 107.36 |
|  | Data Scientist link  (2022) | 78,780 | 94,690 | 103,500 | 49.76 |
|  | Pharmacist link (2023) | 134,450 | 130,510 | 136,030 | 64.81 |
|  | Business Operations Specialists, All Other link  (2023) | 77,110 | 64,380 | 79,590 | 38.26 |
| WashU | Pediatrician, General link  (2023) | 204,120 | 189,930 | 198,690 | 95.53 |
|  | Computer and Information System Managers  link (2024) | Not available | Not available | 171,200  (median) (range not available) | 82.31 |
|  | Pharmacist link (2023) | 134,450 | 130,510 | 136,030 | 64.81 |
|  | Project Management Specialists  link (2023) | 85,660 | 81,170 | 98,580 | 47.39 |

**Supplemental Table 3**. Crosswalk between Low- and High-intensity Intervention Arm Role-and Site-based Personnel Time Costs and Percent Total Contribution

General link (2023)

| Site US Bureau | BLS | Total | Total | Total | Total | Total | Total |
| --- | --- | --- | --- | --- | --- | --- | --- |
| of Labor | Median | Personnel | percent | Personnel | Personnel | percent | Personnel |
| (BLS) | hourly | Time | contributio | Time Cost | Time | contribut | Time Cost |
| Statistics^16^ | wage ($) | (hours) | n to Low- | Low- | (hours) | ion to | High- |
| link by role |  | Low- | intensity | intensity | High- | High- | intensity |
|  |  | intensity | interventio  n (%) | (hours *  median)^a^ | intensity | intensity  intervent | (hours *  median)^a^ |
|  |  |  |  |  |  | ion |  |
| Pediatrician, |  |  |  |  |  |  |  |

General Internal Medicine Physicians link (2023)

VUMC

Data Scientist link

(2022)

Pharmacist link (2023)

Business Operations Specialists, All Other link

(2023)

**Total VUMC**

95.53 27.25 (49.3) 2603.19 32.25 (29.2) 3080.84

107.36 4 (7.2) 429.44 4 (3.6) 429.44

49.76 0 (0) 0 43 (39.0) 2,139.68

64.81 20 (36.2) 1296.20 20 (18.1) 1296.20

38.26 4 (7.2) 153.04 11 (9.98) 420.86

**Personnel Time Costs (hours)**

Pediatrician,

**55.25 (100) 4481.87 110.25 (100)** **7367.02**

General link (2023)

Computer and Information System Managers

WashU

| link (2024) |  | |
| --- | --- | --- |
| Pharmacist link | 64.81 | 1.75 |
| (2023) |  |  |
| Project |  |  |
| Management |  |  |
| Specialists link | 47.39 | 10 |
| (2023) |  |  |

95.53 14 (50.4) 1337.42 97 (81.7) 9266.41

82.31 2 (7.2) 164.62 2 (1.7) 164.62

(6.3) 113.42 1.75 (1.5) 113.42

18

(36.0) 472.90 (15.2) 853.02

**TOTAL WashU Personnel Time Costs (hours)**

**27.75**

**(100)**

**2088.36**

**118.75**

**(100) 10397.47**

^a^Total median values may differ from actual total values due to rounding error

**Supplemental Table 4.** Blank Cost Template Worksheet

|  | Person entering (last,  first name) | Date of  action/cost (mm/dd/yy) | WHAT  was the action? Please provide  specifics (free text) | What was the relative LIFT of this action? (high,  medium, low) | HOURS  of action required to perform  the task? (number) | HOW OFTEN  (how many times was the task performed? E.g., one-time task versus a repeated task)  (number) | WHO  Completed the action?(can involve multiple  people- list names) | Specifics  of action: (free text) | Additional  Comments: (free text) |
| --- | --- | --- | --- | --- | --- | --- | --- | --- | --- |
| **Clinician Champion Identification** |  |  |  |  |  |  |  |  |  |
| **Patient-Facing Educational Resource Adaptation and**  **Dissemination** |  |  |  |  |  |  |  |  |  |
| Adapt patient-facing open-access educational  resources for site |  |  |  |  |  |  |  |  |  |
| Distribute patient-facing material to clinics (electronic or print as  applicable) |  |  |  |  |  |  |  |  |  |
| Paper and printing costs |  |  |  |  |  |  |  |  |  |
| (Itemize other actions related to Patient-Facing Educational Material Adaptation and Distribution here, including non-personnel costs; add as  many rows as needed) |  |  |  |  |  |  |  |  |  |
| **Clinician-Facing Educational Resource Adaptation, Dissemination, and Credit**  **Tracking** |  |  |  |  |  |  |  |  |  |
| Adapt clinician-facing open-access educational  resources for site |  |  |  |  |  |  |  |  |  |
| Distribute clinician-facing open-access educational resources to  clinics (electronic or |  |  |  |  |  |  |  |  |  |

| print as applicable) |  |  |  |  |  |  |  |  |  |
| --- | --- | --- | --- | --- | --- | --- | --- | --- | --- |
| Market clinician learning sessions for live participation including distribution of clinical education session information to clinics for display (e.g., brochures/poste rs) and notification of MOC2/CME  opportunity |  |  |  |  |  |  |  |  |  |
| Disseminate invitation to  view recorded sessions |  |  |  |  |  |  |  |  |  |
| Track and attest to learning session participation for MOC2/CME  credit |  |  |  |  |  |  |  |  |  |
| Notify clinicians about MOC4/ABFM  PI opportunity and steps to  qualify for credit |  |  |  |  |  |  |  |  |  |
| Track and attest to site-specific clinician participation in performance improvement activities for  MOC4 credit |  |  |  |  |  |  |  |  |  |
| Paper and printing costs |  |  |  |  |  |  |  |  |  |
| (Itemize other actions related to Clinician-Facing Educational Material Adaptation and Distribution here, including non-personnel costs; add as many rows as  needed) |  |  |  |  |  |  |  |  |  |
| **Electronic Health Record (EHR)**  **Prescription Field Modification**  **and Activation** |  |  |  |  |  |  |  |  |  |

| Ensure approval for  EHR changes |  |  |  |  |  |  |  |  |  |
| --- | --- | --- | --- | --- | --- | --- | --- | --- | --- |
| Oversee on-site IT build with modification of prescription fields for antibiotics: Modify prescription quick select duration buttons to show 5 and 10 days; add help text to prescriptions; add hyperlink to local care  guidelines |  |  |  |  |  |  |  |  |  |
| Reset provider “favorites” in EPIC for relevant antibiotic  prescriptions |  |  |  |  |  |  |  |  |  |
| Ensure that modified prescription fields are active  for clinicians |  |  |  |  |  |  |  |  |  |
| Ensure availability of patient-facing infographics in languages appropriate for pt. population for clinician addition into after-visit  summaries |  |  |  |  |  |  |  |  |  |
| Notify clinicians of EHR changes |  |  |  |  |  |  |  |  |  |
| Provide associated and ongoing training, facilitation, and  technical assistance |  |  |  |  |  |  |  |  |  |
| (Itemize other actions related to EMR Modifications here, including non-personnel costs; add as many rows as  needed) |  |  |  |  |  |  |  |  |  |
| **Individualized Audit and Feedback System**  **Creation and Activation** |  |  |  |  |  |  |  |  |  |

| Ensure OASIS code in place at  site |  |  |  |  |  |  |  |  |  |
| --- | --- | --- | --- | --- | --- | --- | --- | --- | --- |
| Beta test OASIS  reporting on-site |  |  |  |  |  |  |  |  |  |
| Ensure system in place to distribute ongoing quarterly OASIS  generated reports to  clinicians |  |  |  |  |  |  |  |  |  |
| Notify clinicians to anticipate audit  and feedback reports |  |  |  |  |  |  |  |  |  |
| Ensure site-specific mechanism to track “read receipts” for  reports |  |  |  |  |  |  |  |  |  |
| Provide associated and ongoing training, facilitation, and  technical assistance |  |  |  |  |  |  |  |  |  |
| (Itemize other actions related to Audit and Feedback Reporting here, including non-personnel costs; add as many  rows as needed) |  |  |  |  |  |  |  |  |  |
| **General Costs** |  |  |  |  |  |  |  |  |  |
| Inform local opinion leaders and leadership |  |  |  |  |  |  |  |  |  |
| Coordinate implementation  of interventions |  |  |  |  |  |  |  |  |  |
| Inform and train clinicians on the  intervention |  |  |  |  |  |  |  |  |  |
| Ongoing training, facilitation, and technical  assistance |  |  |  |  |  |  |  |  |  |
| Recruit clinics  to participate in the intervention |  |  |  |  |  |  |  |  |  |
| Other Intervention Costs (e.g. Clinician  Champion time, etc.) |  |  |  |  |  |  |  |  |  |
